# Flow cytometry enables rapid evaluation of novel, new and niche antimicrobial agents

**DOI:** 10.3389/fmicb.2026.1817087

**Published:** 2026-05-04

**Authors:** Emily Salisbury, Kieran Mulroney, Malgorzata K. Kopczyk, Teagan Paton, Christine F. Carson, Wai Shaun Ho, Aron Chakera

**Affiliations:** 1 School of Medicine, The University of Western Australia, Crawley, WA, Australia; 2 Harry Perkins Institute of Medical Research, Nedlands, WA, Australia; 3 Department of Renal Medicine, Sir Charles Gairdner Hospital, Nedlands, WA, Australia

**Keywords:** antibiotic resistance, antimicrobial susceptibility testing, flow cytometry, novel antibiotic, rapid diagnostics

## Abstract

The global rise of multidrug-resistant bacteria necessitates the development of new antimicrobials and faster diagnostic tools. Conventional antimicrobial susceptibility testing is slow, relying on culture-based methods that delay effective treatment, often with fatal consequences in severe infections. In this study, we evaluate flow cytometry as a rapid, culture-minimal method to assess bacterial responses to six antimicrobials: ceftazidime-avibactam, meropenem-vaborbactam, cefiderocol, doxycycline, omadacycline, and lefamulin. Across 165 evaluable antibiotic-isolate combinations, essential agreement between flow cytometry and broth microdilution minimum inhibitory concentrations was 90.71%. Assessable categorical agreement, determined using the European Committee on Antimicrobial Susceptibility Testing and Clinical and Laboratory Standards Institute breakpoints, was 92.59% for doxycycline, 91.67% for omadacycline, and 100% for meropenem-vaborbactam. Cefiderocol exposure was associated with substantial cell elongation, demonstrating cellular-level antimicrobial effects observed using confocal microscopy and imaging flow cytometry. These findings demonstrate the potential of flow cytometry for novel antimicrobial evaluation, offering rapid insights into drug efficacy with potential to improve clinical outcomes in patients.

## 1 Introduction

Drug-resistant bacterial infections are associated with 4.95 million deaths annually, a number projected to rise to 10 million by 2050 (O’Neill, 2016; World Health Organization [WHO], 2019). Poor antimicrobial stewardship contributes to an increase in multidrug-resistant (MDR) and extensive drug-resistant (XDR) bacterial infections (World Health Organization [WHO], 2019). The World Health Organization (WHO) has classified *Enterococcus faecium*, *Staphylococcus aureus*, *Klebsiella pneumoniae*, *Acinetobacter baumannii*, *Pseudomonas aeruginosa*, and *Enterobacter* spp. (ESKAPE pathogens) as top priority pathogens due to their increased resistance and prevalence in healthcare settings, particularly among immunocompromised patients (World Health Organization [WHO], 2024). They display increased resistance in complicated urinary tract infections (cUTI), hospital-acquired bacterial pneumonia (HABP) and intra-abdominal infections, along with *Escherichia coli* (Murray et al., 2022). The emergence of extended spectrum beta lactamases (ESBLs) conferring resistance to penicillins, third generation cephalosporins and monobactams, and carbapenemases, has rendered many existing antibiotics ineffective. This necessitates the development of novel antimicrobials as well as re-evaluation of existing antibiotics with activity against these resistant pathogens.

One approach to overcome beta-lactamase mediated resistance is via the combination of beta-lactams and beta-lactamase inhibitors (BLIs). Beta-lactam and BLI combination antibiotics bind to penicillin binding proteins (PBPs) to prevent cell wall synthesis, while also preventing inactivation of the drug by beta-lactamase producing bacteria (Cook and Wright, 2022; Miller and Arias, 2024). Novel beta-lactam and BLI combination antibiotics include meropenem-vaborbactam (MVB) and ceftazidime-avibactam (CZA). Meropenem-vaborbactam is effective in the treatment of serious Gram-negative infections caused by *Klebsiella pneumoniae* carbapenemase-producing organisms (KPCs) and other carbapenem-resistant Enterobacterales (CREs), which are implicated in cUTI and pyelonephritis (Lomovskaya et al., 2017; Bhowmick and Weinstein, 2020). Ceftazidime-avibactam is used to treat cUTI, intra-abdominal infections and HABP (Lomovskaya et al., 2017; Bhowmick and Weinstein, 2020; Kanj et al., 2022). Other newer antimicrobial agents, such as cefiderocol, naturally capture iron for bacterial uptake and have been utilized as a vehicle for efficient intracellular antibiotic delivery (McCreary et al., 2021; Cook and Wright, 2022; García-Castro et al., 2023). Cefiderocol inhibits bacterial cell wall synthesis through binding penicillin binding protein 3 and is approved for the treatment of CRE infections (McCreary et al., 2021).

Specific challenges posed by emerging resistance mechanisms are also addressed in part by re-visiting older agents. Omadacycline and doxycycline are bacteriostatic tetracyclines that inhibit bacterial replication by binding to the 30S ribosomal subunit (Fluit et al., 2019; Ghatak and Holland, 2023). Although not a novel agent, doxycycline has re-emerged as an option to combat MDR Gram-negative organisms (De Macedo et al., 2023). Newer agents from older classes also help address specific contemporary resistance issues. Lefamulin, a pleuromutilin antibiotic, targets the 50S ribosomal subunit to achieve the same effect (Adhikary et al., 2022; Butler et al., 2023). It is used to treat community-acquired bacterial pneumonia (CABP) and exhibits activity against Gram-positive pathogens (Fluit et al., 2019; Butler et al., 2023).

Accurate and rapid antimicrobial susceptibility testing (AST) is essential for evaluating antibiotic efficacy and guiding prompt, effective clinical therapy. Conventional manual AST, such as broth microdilution (BMD) or agar dilution methods, are culture or growth-based, relying on extended incubation for results (Marutescu, 2023; Salam et al., 2023). Automated AST instrumentation, such as the VITEK^®^ 2 (BioMerieux, France) and BD Phoenix (Becton Dickinson, New Jersey, United Statea) are used in large-scale laboratories and allow for simultaneous isolate testing, decreasing time to result (Kaprou et al., 2021; Salam et al., 2023). However, novel antimicrobials are not available for use on these automated platforms until they gain regulatory market authorization, restricting AST options to slow, conventional methods. Patients with severe bloodstream infections rely on timely and effective antibiotic therapy to appropriately target the causative pathogen, with each hour of antibiotic delay increasing mortality by 7% (Kumar et al., 2006). In recent years, flow cytometry has emerged as a powerful tool for antimicrobial testing, offering rapid, single-cell resolution analysis of bacterial viability, with susceptibility results available in as little as 2 h (Marutescu, 2023). In this study we explored a flow cytometric-based method in the rapid evaluation of susceptibility to novel antimicrobials; ceftazidime-avibactam (CZA), meropenem-vaborbactam (MVB), cefiderocol (FDC), doxycycline (DOX), omadacycline (OMC), and lefamulin (LEF).

## 2 Materials and methods

### 2.1 Bacterial isolates

Control isolates from the American Type Culture Collection [ATCC], 2022 included ATCC 25922 *E. coli*, ATCC 27853 *P. aeruginosa*, ATCC 700603 *K. pneumoniae*, ATCC BAA-1705 *K. pneumoniae* and ATCC 29213 *S. aureus*. Clinical isolates were selected based on historical AST and genotyping results provided by the reference laboratory (PathWest Laboratory Medicine WA, Perth, Australia). To highlight the effect of beta-lactamase inhibitors on meropenem and ceftazidime, four susceptible and four resistant isolates were tested, with resistance mechanisms aligning with the intended target of the novel agents, as shown in Supplementary Table 1. Breakpoints, where available, were applied using both the Clinical and Laboratory Standards Institute (CLSI) Performance Standards for Antimicrobial Susceptibility Testing, M100 35*^th^* edition (2023) and the European Committee on Antimicrobial Susceptibility Testing (EUCAST) breakpoint tables for interpretation of MICs and zone diameters, version 15 (2025). Bacterial isolates were stored in brain heart infusion broth with 15% glycerol (PathWest Media, PathWest Laboratory Medicine WA, Perth, Australia) at −80 °C and recovered as per the ATCC Bacterial Culture Guide (2022).

### 2.2 Antimicrobial plate preparation

Lyophilized antimicrobials were reconstituted to prepare the following stock solutions: 400 mg/L of avibactam in de-ionized water; 100 mg/L of cefiderocol (Sigma-Aldrich, Missouri, United States) in dimethyl sulfoxide (DMSO; Sigma-Aldrich, Missouri, United States); 8,000 mg/L of ceftazidime (Sigma-Aldrich, Missouri, United States) in de-ionized water; 10,000 mg/L of doxycycline (Sigma-Aldrich, Missouri, United States) in de-ionized water; 100 mg/L of lefamulin (Sigma-Aldrich, Missouri, United States) in DMSO; 8,000 mg/L of meropenem (Sigma-Aldrich, Missouri, United States) in de-ionized water; 800 mg/L of omadacycline (Sigma-Aldrich, Missouri, United States) in DMSO; and 800 mg/L of vaborbactam (Sigma-Aldrich, Missouri, United States) in DMSO. Stocks prepared in de-ionized water were syringe filtered at 0.22 μm and subsequently stored at −80 °C. Cefiderocol was diluted from stock in iron-depleted cation adjusted Mueller Hinton broth (ID-CAMHB) prepared using a previously described method (Hackel et al., 2019). All other antibiotics were diluted in cation-adjusted, 0.22 μm filtered Mueller Hinton broth (MHB - Oxoid, Basingstoke, England). For antibiotics dissolved in DMSO, it was ensured that the final concentration of DMSO exposed to bacteria was <1%. To prepare the antimicrobial plates, 50 μl of 2-fold serially diluted antimicrobials were dispensed in duplicate 96 well U-bottom plates (Greiner Bio-One, Kremsmünster, Austria) for flow-cytometric analysis and BMD comparison. Antimicrobial concentration ranges were determined based on MIC distributions and quality control (QC) ranges recommended by the CLSI, M100 35*^th^* edition (2023) and EUCAST breakpoint tables for interpretation of MICs and zone diameters, version 15 (2025), to generate an on-scale phenotype (Supplementary Table 2).

### 2.3 Antimicrobial exposure

Bacterial isolates were incubated at 35 °C overnight in tryptone soya broth (TSB; PathWest Media, PathWest Laboratory Medicine WA, Perth, Australia). These cultures were diluted 1:1000 in Hanks balanced salt solution (HBSS – PathWest Media, PathWest Laboratory Medicine WA, Perth, Australia) and stained with SYTO^®^ 9 (Thermo Fisher Scientific, Massachusetts, United States) for 10 min. To stain bacteria with SYTO^®^ 9, 1 mL was added to each milliliter of diluted culture to achieve a final dye concentration of 5 μM. Suspension density was determined using the Attune™ Cytpix flow cytometer with an Attune™ NxT autosampler (Thermo Fisher Scientific, Massachusetts, United States). Isolates were then standardized to 1 × 10^6^ cells/mL in 6 mL of MHB. A total of 50 μL of this suspension was overlayed into each well of the prepared antimicrobial plates; one plate for flow-cytometric analysis and one for BMD comparison. All samples were processed in triplicate columns. Immediately upon inoculation of the plates, baseline (T0) cell counts were prepared using a 1:1 ratio of the standardized bacterial suspension and sterile HBSS. These were stained with SYTO^®^ 9 (as previously described) to measure the inoculum density of cells in the initial suspension and an unstained control distinguished true-positive stained cells from background fluorescence. Both plates were incubated at 35 °C, with the flow cytometer plate analyzed after 3 h of incubation while the BMD plate was read after 24 h. Plates assessed using the flow cytometer were stained with SYTO^®^ 9 nucleic acid stain (in HBSS) for 10 min and analyzed using the Attune™ Cytpix. BMD plates were visualized using the Sensititre Vizion Digital MIC Viewing System (Thermo Fisher Scientific, Massachusetts, United States).

### 2.4 Flow cytometer settings

The Attune™ Cytpix flow cytometer was configured with a 561 nm yellow laser (YL1: 585/16 nm, YL2: 620/15 nm, YL3: 780/60 nm), a 488 nm blue laser (BL1: 530/30 nm, BL2: 695/40 nm), a 637 nm red laser (RL1: 670/14 nm, RL2: 720/30 nm, RL3: 780/60 nm) and a 405 nm violet laser (VL1: 450/40 nm, VL2: 525/50 nm, VL3: 610/20 nm, VL4: 660/20 nm, VL5:710/50 nm, VL6: 780/60 nm). Voltages for SYTO^®^ 9-stained Gram-negative isolates were: Forward scatter (FSC) 340 (Threshold 0.7 × 1000 AND), side scatter (SSC) 360 (Threshold 0.2 × 1000 AND), BL1 260 (Threshold 0.1 × 1000 AND), BL2 300, RL1-3 300, VL1-6 400, YL1-3 300. SYTO^®^ 9-stained Gram-positive isolate voltages were: FSC 280 (Threshold 0.5 × 1000 AND), SSC 300 (Threshold 0.1 × 1000 AND), BL1 280 (Threshold 0.1 × 1000 AND), BL2 300, RL1-3 300, VL1-6 400M YL1-3 300. Unstained samples were run at the same voltages, with the BL1 threshold set to OFF. A total of 125 μL of sample was acquired with a total sample volume of 200 μL, measured at 200 μL per minute, stopping at 28 μL with one mix and one rinse between wells. Images were taken every 100 events for a total of 500 images, with the camera positioned at 152 width and 248 height (pixels), −1 focus and 43% illumination. Data were exported and analyzed using FlowJo v10.8.1 (BD Life Science, Ashland, United States) and GraphPad Prism v10.2.3 (GraphPad Software, San Diego, United States).

### 2.5 Gating strategy

**FIGURE 1:**
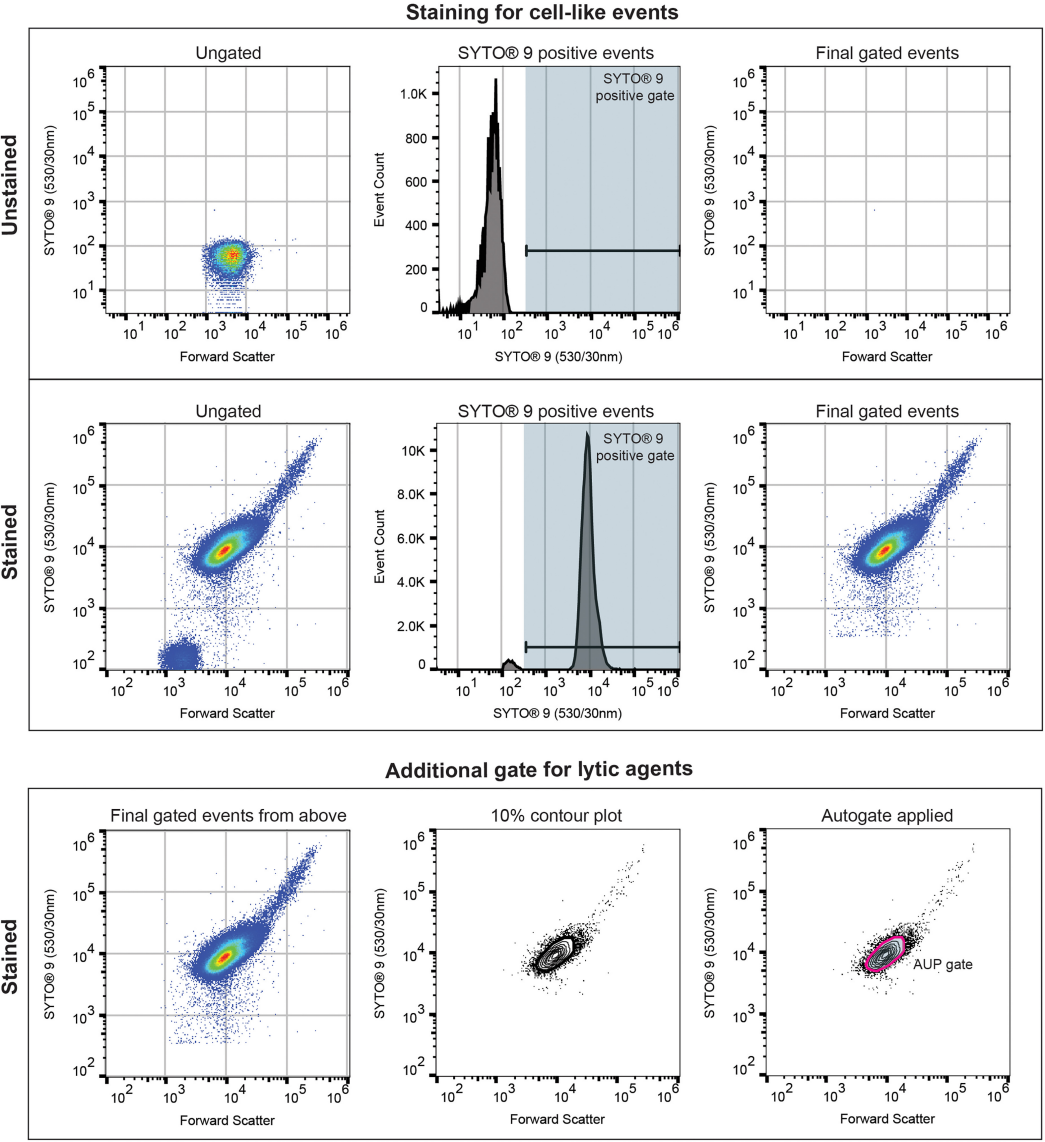
Flow cytometry gating strategy optimized to detect cell-like events, an example using ATCC 25922 (*E. coli*). The SYTO^®^ 9 positive event gate is set at the upper limit of the unstained control to detect cell-like events and is applied to all isolates tested. Ceftazidime-avibactam and meropenem-vaborbactam require the addition of the antimicrobial unexposed population (AUP) gate to remove lysed cells for analysis. The AUP gate is determined using the FlowJo Autogate tool on a 10% contour plot.

All isolates were gated using a SYTO^®^ 9 positive histogram gate, which was set at the upper limit of the unstained control and applied to all other replicates of the same isolate to exclude machine and background noise (Figure 1). Lytic agents such as CZA, MVB, and FDC required further gating to remove lysed cell debris for analysis. For these agents, we adopted a previously described method by Mulroney et al. (2017), using FlowJo, to apply a 10% contour plot on the FSC vs SYTO^®^ 9 subset of cells that were SYTO^®^ 9 positive. This contained 90% of the SYTO^®^ 9 positive cells. The Autogate tool allowed for selection of this population, referred to as the antimicrobial unexposed population (AUP).

### 2.6 Flow cytometry analysis

A set of rules were defined for the evaluation of bacterial isolates using flow cytometry. To analyze the data for predicted MIC determination, tables exported from FlowJo included SYTO^®^ 9 gate counts (or AUP gate for lytic agents), sample volume and median BL1 (SYTO^®^ 9) and FSC values. Bacterial doublings were calculated for each isolate by determination of the event count increase from T0 counts, transformed to log_2_. This allowed for approximation of binary fission, quantifying the number of cell divisions, and predicted bacteriostasis when doublings cease. Bacterial counts were first converted to cell suspension densities and normalized to the unexposed control, expressed as a percentage. The relative change in bacterial growth was then expressed as a delta (Δ) count ratio. A threshold of Δ ≤ 10% was applied based on validated assumptions from previous work (Mulroney et al., 2017, 2022). For the lowest drug concentration, the delta count ratio was calculated as the normalized value minus 100% (the control baseline). For all higher concentrations, the delta ratio was calculated as the normalized value at that concentration minus the normalized value at the immediately preceding concentration. The MIC was predicted to be the concentration where the following criteria were met: (1). Bacterial doublings had ceased, as indicated by an error in log transformation of the counts. (2). The Δ count ratio was ≤10%.

To quantify the antibiotic-induced shifts in response to addition of beta-lactamase inhibitors, we also calculated the Δ median of both FSC and SYTO^®^ 9 fluorescence intensity. This represented changes in central tendency relative to the unexposed control. Flow cytometry MICs were compared with BMD MICs to calculate variance for essential and categorical agreement. Essential agreement (EA) is defined as agreement in MIC within one doubling dilution of a test method compared to a reference method (Clinical and Laboratory Standards Institute [CLSI], 2015). Assessable EA was evaluated for all MICs within the measurable concentration range; values falling outside the range (greater than the maximum concentration tested) were excluded. Where appropriate, categorical agreement (CA) was determined using the corresponding SIR interpretations from Clinical and Laboratory Standards Institute [CLSI] (2023) and The European Committee on Antimicrobial Susceptibility Testing [EUCAST] (2025) breakpoint tables. Assay performance was assessed according to the performance criteria for reference and new antimicrobial susceptibility testing methods defined by the U.S. Food and Drug Administration (2009), with categorical and essential agreement of >89.9% considered acceptable. Where no resistant isolates were tested, CA was not calculated. EUCAST breakpoints were not available for doxycycline or omadacycline, and CLSI breakpoints were not available for lefamulin; these agents were therefore excluded from their respective analyses.

### 2.7 Quality control and exclusion criteria

Antimicrobial plates were quality controlled against ATCC control strains as recommended by Clinical and Laboratory Standards Institute [CLSI] (2023) and The European Committee on Antimicrobial Susceptibility Testing [EUCAST] (2025). To do this, BMD plates were prepared for each control strain, as previously described, to ensure the QC target was met. To confirm by classical methods that the suspension density was correct for BMD and flow cytometric assays and to assess purity of the culture, colony counts were performed on inoculum suspensions. A 1:250 dilution of the standardized bacterial suspension (1 × 10^6^ cells/mL) was prepared in sterile HBSS. A total of 10 μL was plated on blood agar, yielding an expected inoculum of 40 CFU per plate. Plates were incubated at 35 °C and colonies counted 24 h later. Colony counts within ±0.5 log_10_ were considered acceptable, consistent with CLSI recommendations for inoculum verification (Clinical and Laboratory Standards Institute [CLSI], 2024). Following analysis, isolates were evaluated for quality control. For an organism to be valid for flow cytometry MIC determination, at least one cell division must have occurred from T0 baseline counts, following incubation with an antimicrobial agent. This is to appropriately analyze cell wall disruptive antimicrobial agents (e.g., beta-lactams) which only impact cells actively dividing. CRE5 and CRE14 (cefiderocol) and WGS7 (omadacycline) were excluded from analysis as the MICs generated were off-scale. UPS11 (cefiderocol) was also excluded for lack of active bacterial division.

### 2.8 Confocal microscopy

Flow cytometric counts were performed, as described above, on the overnight TSB bacterial suspension to adjust bacterial concentrations to 1 × 10^6^ cells/mL in 1 mL of MHB. This suspension was mixed with 1 mg/L cefiderocol for final concentrations of 0.5 mg/L and 5 × 10^5^ cells/mL. All tubes were incubated at 35 °C for 3 h before centrifugation at 7,800 × *g* for 5 min. The pellet was resuspended in 10 μL of HBSS, stained with SYTO^®^ 9 (as described previously). A 2 μL drop was placed on a poly-L-lysine slide (ProSciTech, Townsville, Queensland) and allowed to air dry before sealing with a coverslip (Thermo Fisher Scientific, Massachusetts, United States). Slides were observed under a 20x objective (numerical-aperture 0.75) using a Nikon A1RMP confocal microscope (488 nm laser - Nikon, Tokyo, Japan) with a digital zoom of 2 × applied. Images were collected using the NIS Elements Viewer v5.22.

## 3 Results

**FIGURE 2:**
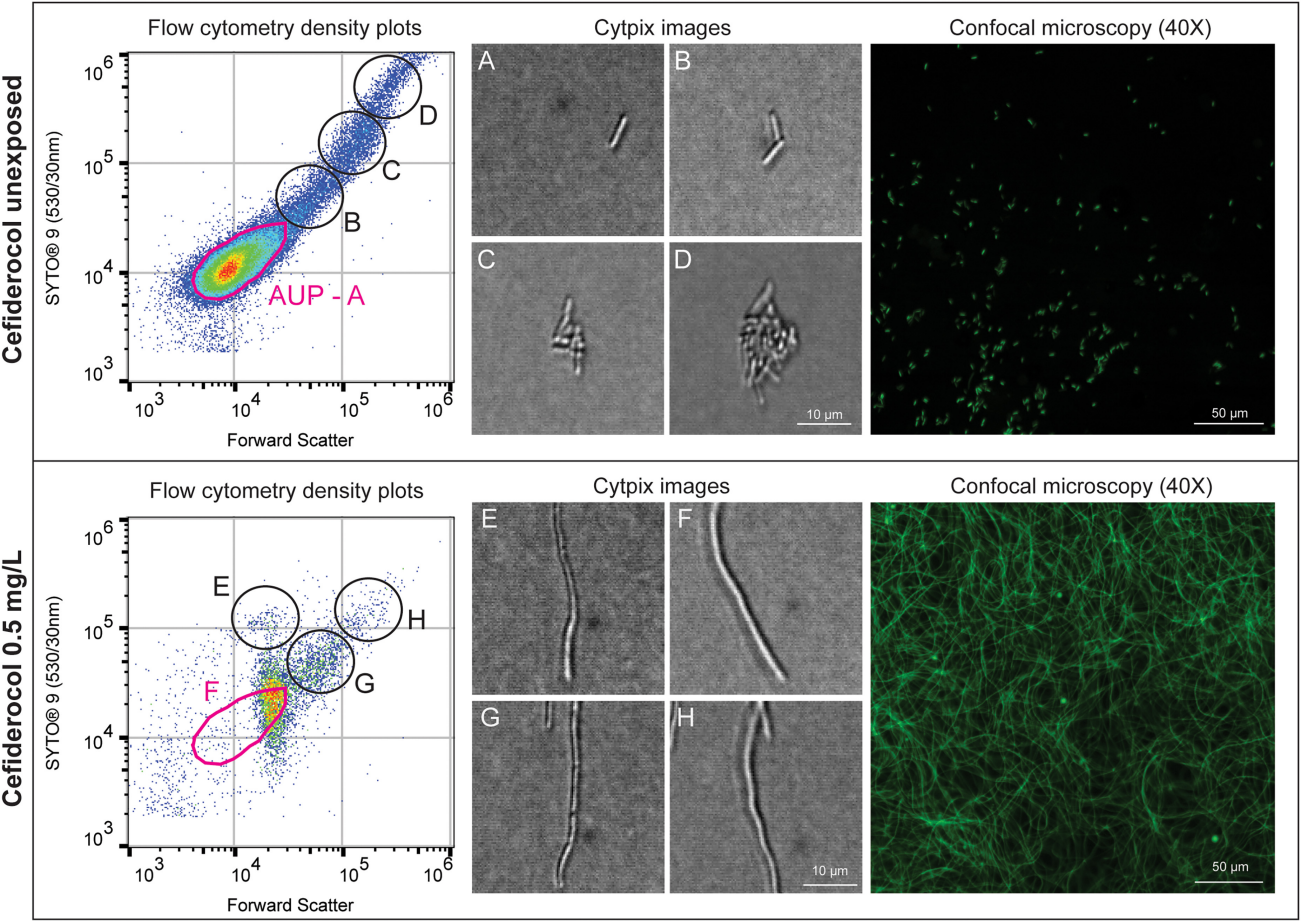
Phenotypic effects of cefiderocol on ATCC 25922 *E. coli* at the unexposed concentration (0 mg/L) and broth microdilution (BMD) minimum inhibitory concentration (MIC) (0.5 mg/L). SYTO^®^ 9 gated events (left) highlight population distributions, with aggregation observed at higher forward scatter and SYTO^®^ 9 fluorescence in the unexposed sample, as shown in Attune™ Cytpix images **(A–D)**. Attune™ Cytpix images (center) and confocal microscopy (right) reveal cell wall structural changes, with elongation visible in the presence of 0.5 mg/L cefiderocol **(E–H)**.

For cefiderocol, in the unexposed control, ATCC 25922 *E. coli* is observed with multiple population distributions (Figures 2A–D). Non-aggregated bacterial cells in the AUP (Figure 2A) were observed with lower forward scatter and SYTO^®^ 9 fluorescence levels compared to highly aggregated populations (Figure 2D) which were higher in forward scatter and SYTO^®^ 9 fluorescence. When exposed to 0.5 mg/L cefiderocol (the BMD MIC), cell elongation is evident in both the Attune™ Cytpix images and confocal microscopy (Figures 2E–H), with a 97% reduction of cell events within the AUP gate.

**FIGURE 3:**
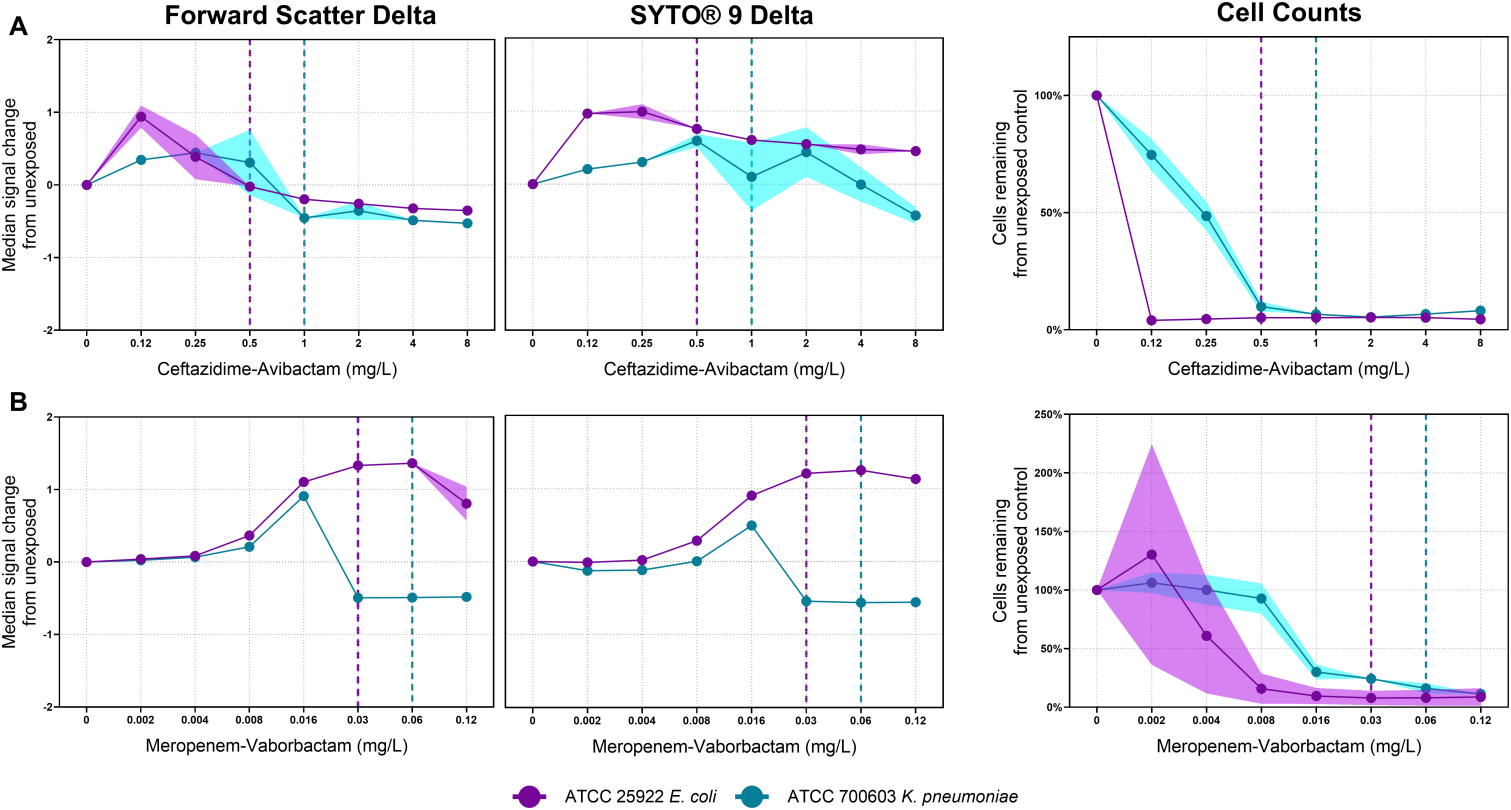
Median signal change of forward scatter and SYTO^®^ 9 fluorescence from the unexposed control in ATCC 25922 *E. coli* (purple) and ATCC 700603 *K. pneumoniae* (blue), exposed to ceftazidime-avibactam **(A)** and meropenem-vaborbactam **(B)**. Shaded error bars represent standard deviation. Dotted lines represent the minimum inhibitory concentration determined by broth microdilution for each isolate.

ATCC 700603 and ATCC 25922 were used as control isolates to demonstrate differential responses to beta-lactam-beta-lactamase inhibitor combinations (Figure 3). For ATCC 700603, exposure to both ceftazidime-avibactam and meropenem-vaborbactam resulted in an initial upward shift in FSC and SYTO^®^ 9 fluorescence median Δ associated with increasing antibiotic concentration. This was followed by a subsequent decline in both FSC and SYTO^®^ 9 fluorescence, indicating cell lysis. For ceftazidime-avibactam (Figure 3A), median Δ values shifted from 0.31 to −0.45 (FSC) and from 0.59 to 0.10 (SYTO^®^ 9) at 0.5 and 1 mg/L, respectively. Similarly, with meropenem-vaborbactam (Figure 3B), FSC median Δ decreased from 0.91 to −0.49 and SYTO^®^ 9 from 0.49 to −0.54 at 0.016 and 0.03 mg/L, respectively. In contrast, ATCC 25922 exhibited only modest changes, with median Δ values plateauing rather than declining.

Table 1 outlines MIC values and corresponding interpretation data for resistant isolates exposed to meropenem and ceftazidime, with and without addition of beta-lactamase inhibitors (avibactam and vaborbactam, respectively). For resistant isolates exposed to ceftazidime: LGC7 (MIC 512 mg/L), LGC13 (256 mg/L), LGC23 (32 mg/L), and LGC70 (>512 mg/L) – the addition of avibactam reduced MICs to <8 mg/L, restoring susceptibilities according to both EUCAST and CLSI criteria. Similarly, meropenem MICs for KPC-producing isolates LGC7 and LGC70 decreased from 64 to 1 mg/L, and for CRE1 [*OXA-1*, *TEM*, *CTX-M-15*, *TET(A*)], from 2 to 0.25 mg/L following the addition of vaborbactam. In contrast, CRE14 (*OXA-181*, *CTX-M-15*) retained a high MIC of 32 mg/L with no change in susceptibility, indicating resistance to both meropenem and meropenem-vaborbactam.

**TABLE 1:** Minimum inhibitory concentration (MIC) of resistant isolates in response to ceftazidime and meropenem exposure, with and without beta-lactamase inhibitors.

Isolate Details				Flow cytometry-derived MIC (mg/L) and interpretation (SIR)					
Isolate	Genus	Species	Resistance genes	Ceftazidime	EUCAST	CLSI	Ceftazidime-avibactam	EUCAST	CLSI
LGC7	*Klebsiella*	*pneumoniae*	*blaOXA, blaTEM-1, blaKPC-2, blaSHV-12, mph(A)*	512	R	R	2	S	S
LGC13	*Klebsiella*	*aerogenes*	*ampC-Kaer*	16	R	R	0.5	S	S
LGC23	*Escherichia*	*coli*	*blaOXA-48, blaDHA-1, bla-CTX-M-27, tet(A), mph(A)*	32	R	R	0.5	S	S
LGC70	*Klebsiella*	*pneumoniae*	*blaOXA, blaTEM-1, blaKPC-2, blaSHV-12, mph(A)*	>512	R	R	2	S	S
**Isolate**	**Genus**	**Species**	**Resistance genes**	**Meropenem**	**EUCAST**	**CLSI**	**Meropenem-vaborbactam**	**EUCAST**	**CLSI**
CRE1	*Escherichia*	*coli*	*blaOXA-1, blaTEM, blaCTX-M-15, tet(A)*	2	S	I	0.25	S	S
CRE14	*Klebsiella*	*pneumoniae*	*OXA-181, CTX-M-15*	32	R	R	32	R	R
LGC7	*Klebsiella*	*pneumoniae*	*blaOXA, blaTEM-1, blaKPC-2, blaSHV-12, mph(A)*	4	I	R	2	S	S
LGC70	*Klebsiella*	*pneumoniae*	*blaOXA, blaTEM-1, blaKPC-2, blaSHV-12, mph(A)*	4	I	R	0 2	S	S

Minimum inhibitory concentrations (MIC) results were obtained using flow cytometric analysis. SIR classification was determined using European Committee on Antimicrobial Susceptibility Testing (EUCAST) and Clinical and Laboratory Standards Institute (CLSI) breakpoints. EUCAST: S, susceptible; I, susceptible, increased exposure; R, resistant. CLSI: S, susceptible; I, intermediate; R, resistant.

**FIGURE 4:**
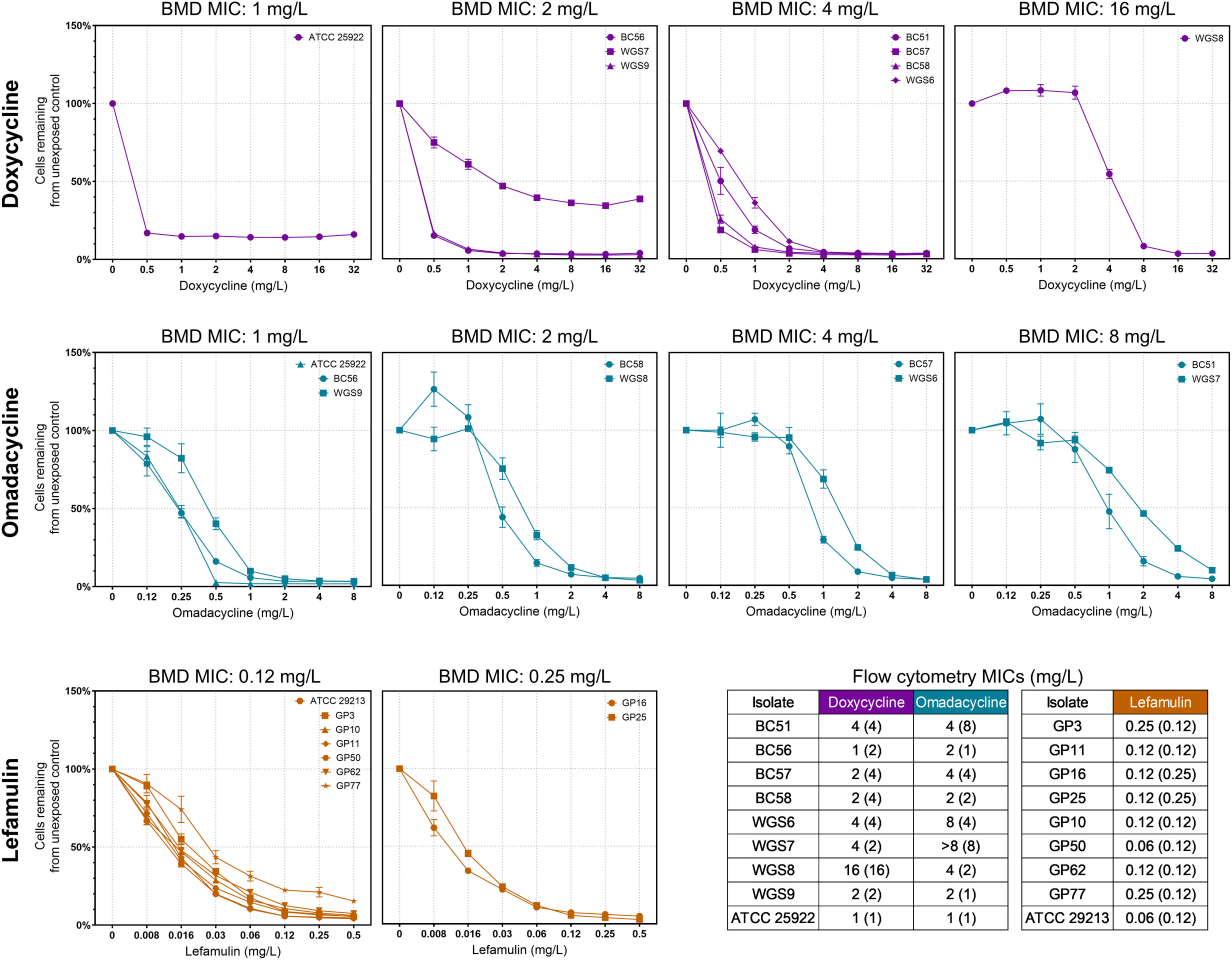
Flow cytometry-derived event count curves for Enterobacterales exposed to non-lytic agents (doxycycline, omadacycline, or lefamulin), stratified by broth microdilution (BMD) minimum inhibitory concentrations (MICs). Relative cell counts represent the proportion of cells remaining relative to the unexposed control. Error bars represent standard deviation. The corresponding flow cytometry MICs are summarized in the adjacent tables to demonstrate essential agreement within ± 1 dilution of the BMD MICs, displayed in brackets.

For non-lytic protein synthesis inhibitors (DOX, OMC, and LEF), a concentration-dependent decline in bacterial events was observed (Figure 4). While these agents did not induce shifts in phenotypic signatures (either by FSC or SYTO^®^ 9) or distinct morphological subpopulations, the reduction in cell counts aligned with reference MICs by BMD. For all three agents, isolates with higher BMD MICs exhibited right-shifted concentration-responses, indicating reduced susceptibility. Despite subtle phenotypic shifts, flow cytometry-derived MICs demonstrate essential agreement with BMD MICs (doxycycline and lefamulin 96.3% EA and omadacycline 100% EA).

**FIGURE 5:**
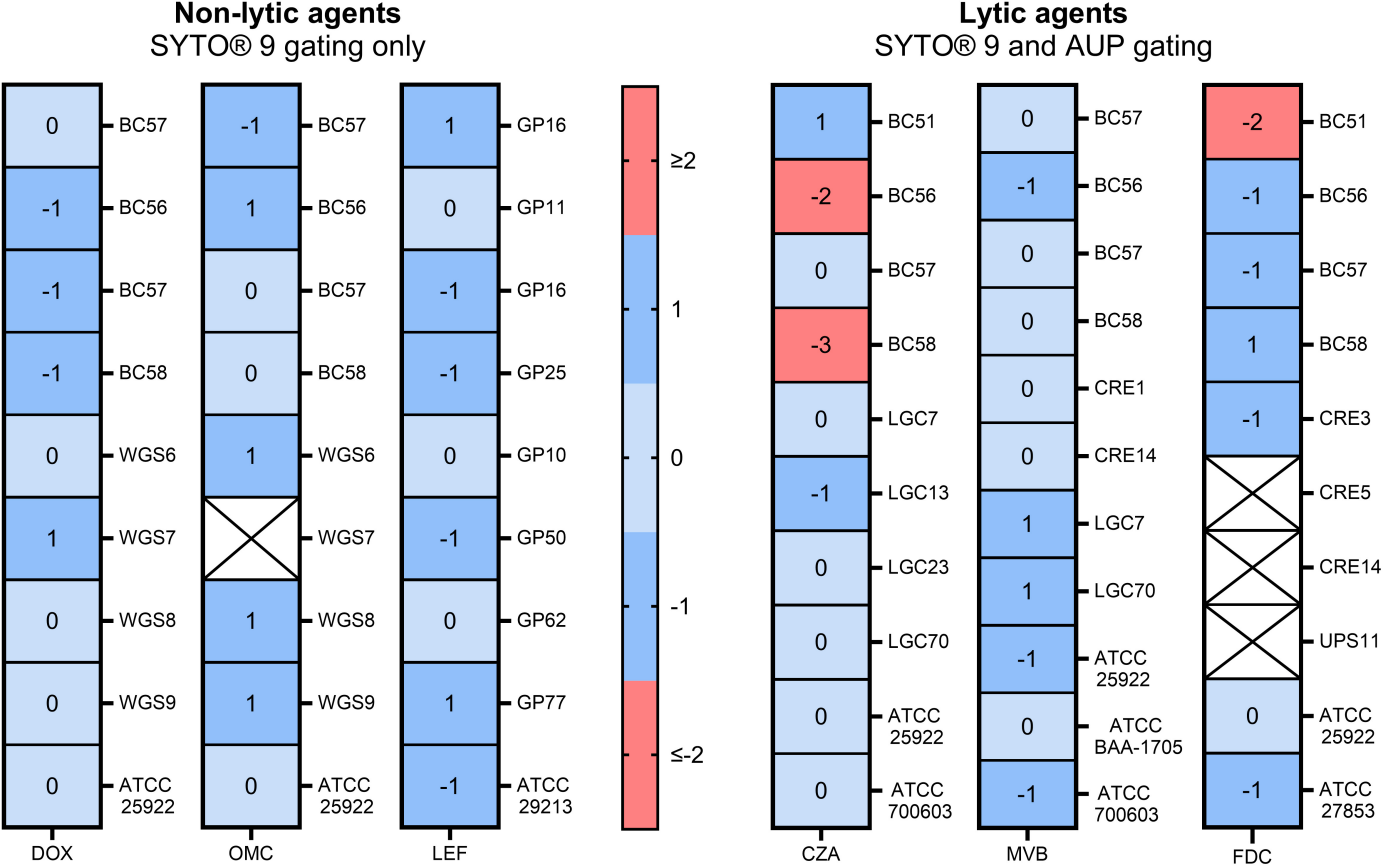
Variance in minimum inhibitory concentrations generated using broth microdilution and flow cytometry. Non-lytic agents (DOX, OMC, and LEF) were analyzed using the SYTO^®^ 9 positive gate only. Lytic agents (CZA, MVB, and FDC) were analyzed using the SYTO^®^ 9 gate and additional antimicrobial unexposed population (AUP) gate to remove lysed cells. Blank values represent isolates excluded from analysis as the minimum inhibitory concentrations (MICs) determined by both methods were off scale (CRE5 and CRE14 for cefiderocol, and WGS7 for omadacycline) or due to the lack of bacterial division following incubation (UPS11 for cefiderocol).

Amongst the 165 on-scale combinations, assessable EA was 90.71% (Figure 5). CA for doxycycline and omadacycline was 92.59% and 91.67%, respectively (interpreted using CLSI breakpoints) and meropenem-vaborbactam was 100% (interpreted using both EUCAST and CLSI breakpoints). BC56 and BC58 (both *E. coli*) exhibited variance ≤ 2 dilutions below the BMD MIC in response to ceftazidime-avibactam. Similarly, BC51 (*E. cloacae*) had a flow cytometry MIC 2 dilutions lower in response to cefiderocol.

## 4 Discussion

### 4.1 Phenotypic investigation of cefiderocol

Consistent with the observations of earlier studies, our study found that real-time imaging using the Attune™ Cytpix revealed cefiderocol-induced elongation in susceptible isolates. Cefiderocol inhibits bacterial cell wall synthesis by binding to key bacterial enzymes necessary for building and maintaining cell wall structure. It preferentially binds PBP3 (and other PBPs), thereby preventing septum formation and cell division (Bao et al., 2022; Ferretti et al., 2024). Ferretti et al. (2024) previously demonstrated that cefiderocol-induced PBP inhibition in *P. aeruginosa* led to the upregulation of the cytoskeletal proteins *MreC* and *MreB*, promoting elongation as observed using scanning electron microscopy. Similarly, Bao et al. (2022) reported filament formation in *K. pneumoniae* following cefiderocol exposure. This supports the utility of flow cytometry as a rapid phenotypic screening tool for antimicrobial activity in drug development activities.

### 4.2 Responses to beta-lactamase inhibitors

Using flow cytometry, ceftazidime-avibactam and meropenem-vaborbactam induced comparable phenotypic signatures in both *E. coli* ATCC 25922 and *K. pneumoniae* ATCC 700603 (Figure 3). Differences in signal intensity between the two isolates may reflect strain-specific responses to beta-lactam and beta-lactamase inhibitor combinations, as ATCC 700603 expresses the ESBL *SHV-18* and ATCC 25922 does not. Variability in residual cell counts for ATCC 25922 was noted, potentially attributed to bacterial aggregation leading to elevated cell counts, as demonstrated in Figure 2. While meropenem and ceftazidime are widely available and have been in use for many years, the combination with beta-lactamase inhibitors results in novel therapeutic activity. To evaluate these combinations, we analyzed flow cytometry-derived signatures to predict MICs with and without avibactam or vaborbactam. CRE1 (*E. coli*) harbored the ESBL genes *OXA-1*, *TEM*, *CTX-M-15*, *and tet(A)*, and displayed a meropenem MIC of 2 mg/L by flow cytometry. This was interpreted as susceptible using EUCAST criteria, suggesting the absence of carbapenemase-mediated resistance. With the addition of vaborbactam, the MIC decreased 8-fold to 0.25 mg/L, likely due to *TEM* inhibition, highlighting the level of detail visible using flow cytometry. In contrast, CRE14 (*K. pneumoniae*) remained resistant to meropenem despite addition of vaborbactam, attributable to the presence of *OXA-181*, a class D carbapenemase that is poorly inhibited by vaborbactam (Lomovskaya et al., 2017; Patrick and Scoble, 2020). These findings demonstrate that flow cytometry can be used not only to evaluate novel antimicrobials, but also to rapidly assess new combinations of existing drugs.

### 4.3 Limitations of the method

To improve essential and categorical agreement for cefiderocol in future analyses, an expanded concentration range is required to better capture resistant phenotypes. A broader dilution series would allow more accurate determination of MIC values for isolates with reduced susceptibility, reducing truncation effects at the upper concentrations. The observed variance of ≤2 dilutions in ceftazidime-avibactam and cefiderocol likely reflects greater initial phenotypic shifts in forward scatter and SYTO^®^ 9 fluorescence, resulting in under-calling of MICs by flow cytometry. With bacterial populations leaving the AUP gate abruptly, cell counts are reduced at sub-inhibitory concentrations. The AUP also precludes analysis of cells that are elongated in response to antimicrobial insult but may be capable of division. Given the volume and complexity of flow cytometry data, these results highlight machine learning approaches as an essential tool for enhancing diagnostic utility and analysis, which could increase throughput in future studies. Machine learning pipelines for AST data have been previously reported to accelerate the analytical process, enabling rapid reporting of results for patients with life-threatening infections (Inglis et al., 2020). Instrument accessibility and costs remain barriers to flow cytometry implementation, particularly in resource-limited settings (Marutescu, 2023). Further validation studies against gold-standard AST methods are necessary to support its broader clinical implementation.

### 4.4 Application of flow cytometry in MIC prediction

Bacterial structural diversity and antibiotic complexity make drug innovation difficult, and in some cases, by the time a novel drug is approved, resistance has already been reported (Lomovskaya et al., 2017; Cook and Wright, 2022; Miller and Arias, 2024). Flow cytometry has emerged as a promising solution to many of the limitations associated with conventional AST, enabling rapid, single-cell analysis of bacterial responses to antimicrobial exposure, without reliance on culture-based growth or molecular genotyping. This phenotypic approach supports quicker MIC determination, yielding results faster for critically ill patients requiring effective antimicrobial intervention.

Antimicrobial resistance is on the rise, with approximately 35% of bacterial infections already displaying resistance to currently available medicines (World Health Organization [WHO], 2019). This impacts not only community health, but also critical care, chemotherapy, post-surgery infection control and other clinical settings. With many existing classes of antibiotics rendered ineffective to pathogenic organisms, including reserve antibiotics such as polymixins, aminoglycosides and tigecycline, there is an urgent need to develop novel agents with improved activity against priority bacterial resistance mechanisms. Rapid diagnostics play a crucial role in the deployment of new antibiotics to correctly identify targeted treatment sooner, limiting the need for empirical therapy to reduce resistance development (O’Neill, 2016). Novel antimicrobials play a vital part in the fight against escalating antimicrobial resistance. Our study demonstrates a rapid alternative to traditional antimicrobial susceptibility testing, with 90.71% essential agreement between flow cytometry and reference broth microdilution methods, and results available in 4 h. By enabling phenotypic assessment at the single-cell level, flow cytometry provides detailed insights into antimicrobial activity that conventional methods and other emerging approaches do not. Further research is required for the clinical adoption of flow cytometry into routine susceptibility testing, including validation across diverse bacterial species and resistance mechanisms. With continued refinement, flow cytometry has the potential to improve both antimicrobial development and clinical diagnostics, delivering faster and more targeted treatment for patients facing multidrug-resistant infections.

## Data Availability

The raw data supporting the conclusions of this article will be made available by the authors, without undue reservation.
