# Supplementary material for: Flow cytometry enables rapid evaluation of novel, new and niche antimicrobial agents
Source: Front Microbiol. 2026 May 4;17:1817087. doi: 10.3389/fmicb.2026.1817087 (PMC13180735; doi:10.3389/fmicb.2026.1817087)
Supplement: Supplementary file 1 [file Table_1.docx]

| **Isolate** | **Genus** | **Species** | **Resistance genes** |
| --- | --- | --- | --- |
| ATCC 25922 | Escherichia | coli | None |
| ATCC BAA-1705 | Klebsiella | pneumoniae | *KPC* |
| ATCC 700603 | Klebsiella | quasipneumoniae | *SHV-18* |
| ATCC 27853 | Pseudomonas | aeruginosa | None |
| ATCC 29213 | Staphylococcus | aureus | None |
| BC51 | Enterobacter | cloacae | *blaACT-17* |
| BC56 | Escherichia | coli | None |
| BC57 | Klebsiella | pneumoniae | *blaSHV-1* |
| BC58 | Escherichia | coli | None |
| UPS11 | Pseudomonas | aeruginosa | None |
| CRE3 | Klebsiella | oxytoca | *IMP-4, CTX-M-15* |
| CRE5 | Escherichia | coli | *NDM-5, CTX-M-15* |
| CRE14 | Klebsiella | pneumoniae | *OXA-181, CTX-M-15* |
| CRE1 | Escherichia | coli | *blaOXA-1, blaTEM, blaCTX-M-15, tet(A)* |
| LGC7 | Klebsiella | pneumoniae | *blaOXA, blaTEM-1, blaKPC-2, blaSHV-12, mph(A)* |
| LGC13 | Klebsiella | aerogenes | *ampC-Kaer* |
| LGC23 | Escherichia | coli | *blaOXA-48, blaDHA-1, bla-CTX-M-27, tet(A), mph(A)* |
| LGC70 | Klebsiella | pneumoniae | *blaOXA, blaTEM-1, blaKPC-2, blaSHV-12, mph(A)* |
| WGS6 | Klebsiella | pneumoniae | *blaSHV-11, blaCTX-M-15* |
| WGS7 | Klebsiella | oxytoca | *blaIMP-4, blaTEM-1, blaOXA-1, blaOXY-1-1, tet(A)* |
| WGS8 | Escherichia | coli | *blaCMY-42, blaTEM-1, blaOXA-181, blaCTX-M-15, tet(A)* |
| WGS9 | Klebsiella | oxytoca | *blaTEM-1, blaOXY-1-1, blaIMP-4* |
| GP3 | Staphylococcus | aureus | *blaI, blaR1, blaZ, tet(38)* |
| GP11 | Staphylococcus | aureus | *blaI, blaR1, blaZ, tet(38)* |
| GP16 | Staphylococcus | aureus | *tet(38)* |
| GP25 | Staphylococcus | aureus | *blaI, blaZ, tet(38)* |
| GP10 | Staphylococcus | aureus | *blaZ, blaR1, blaI, mecA, mecR1, tet(38)* |
| GP50 | Staphylococcus | aureus | *blaI, blaR1, blaZ, tet(38)* |
| GP62 | Staphylococcus | aureus | *blaZ, blaR1, blaI, mecA, tet(38), tet(K)* |
| GP77 | Staphylococcus | aureus | *blaR1, blaI, blaPC1, mecA, mecR1, tet(38)* |

**Table S1:** Corresponding resistance genes associated with each isolate tested. Resistance genes were detected using whole genome sequencing and isolates were selected based on resistance mechanisms that aligned with the novel antimicrobials tested.
