# Supplementary material for: Flow cytometry enables rapid evaluation of novel, new and niche antimicrobial agents
Source: Front Microbiol. 2026 May 4;17:1817087. doi: 10.3389/fmicb.2026.1817087 (PMC13180735; doi:10.3389/fmicb.2026.1817087)
Supplement: Supplementary file 2 [file Table_2.docx]

| **Antimicrobials** | **Range tested (mg/L)** | **Isolates tested** |
| --- | --- | --- |
| Cefiderocol | 0.008 - 0.5 | BC51, BC56, BC57, BC58 |
|  | 0.016 - 1 | CRE3, CRE5, CRE14, UPS11, ATCC 25922, ATCC 27853 |
| Ceftazidime | 0.03 - 2 | BC51, BC56, BC57, BC58, ATCC 25922 |
|  | 8 - 512 | LGC7, LGC13, LGC23, LGC70, ATCC 700603 |
| Ceftazidime-avibactam | 0.008 - 0.5 | BC51, BC56, BC57, BC58 |
|  | 0.12 - 8 | LGC7, LGC13, LGC23, LGC70, ATCC 25922, ATCC 700603 |
| Doxycycline | 0.5 - 32 | BC51, BC56, BC57, BC58, WGS6, WGS7, WGS8, WGS9, ATCC 25922 |
| Omadacycline | 0.12 - 8 | BC51, BC56, BC57, BC58, WGS6, WGS7, WGS8, WGS9, ATCC 25922 |
| Lefamulin | 0.008 - 0.5 | GP3, GP11, GP16, GP25, GP10, GP50, GP62, GP77, ATCC 29213 |
| Meropenem | 0.004 - 0.25 | BC51, BC56, BC57, BC58, ATCC 25922, ATCC 700603 |
|  | 1 - 64 | CRE1, CRE14, LGC7, LGC70, ATCC BAA-1705 |
| Meropenem-vaborbactam | 0.002 - 0.12 | BC51, BC56, BC57, BC58, ATCC 25922, ATCC BAA-1705, ATCC 700603 |
|  | 0.016 - 1 | CRE1 |
|  | 1 - 64 | CRE14, LGC7, LGC70 |

**Table S2**: Antimicrobial concentration ranges tested for each isolate to generate on-scale phenotypic signatures. Non-novel comparator antimicrobials are shaded in grey.
